# Translation strategy of legal terms with Chinese characteristics in *Civil Code of the People’s Republic of China* based on Skopos theory

**DOI:** 10.1371/journal.pone.0273944

**Published:** 2022-09-22

**Authors:** Jing An, Jiahui Sun

**Affiliations:** 1 Department of Linguistics, University of California Santa Barbara, Santa Barbara, California, United States of America; 2 School of Juris Master in China University of Political Science and Law, Beijing, China; Victoria University, AUSTRALIA

## Abstract

*The Civil Code of the People’s Republic of China*, known as the “Encyclopedia of social life”, has come into effect since January 1, 2021. It is the first law named after the “Code” and occupies a fundamental position in the China’s legal system. Therefore, translation of legal terms is vital in making target readers familiar with the China’s civil system and contributes to cultural communication and academic exchange. Previous research on translations of legal terms provides various translation principles and strategies. However, culture connotation in Chinese is usually implicit or lacks counterparts, leading to the difficulties in the equivalent translation of legal terms with Chinese characteristics. Therefore, this paper concentrates on the selection of legal term translation principles from the perspective of the Skopos theory and a Chinese translation theory named Eco-translatology, to explore the application and effect of selected principles in the translation of legal terms with Chinese characteristics. Guided by the Skopos theory and Eco-translatology, this paper collects 42 legal terms with Chinese characteristics from the *The Civil Code of the People’s Republic of China* and analyzes the application of 5 translation principles of accuracy, readability, traceability, conventionality, and unification. Through analysis, the legal terms are classified into newly-coined and inherited terms and rules of strategy application are therefore summarized. It is expected that this paper would provide systematic principles for the translation of legal terms with Chinese characteristics.

## Introduction

*The Civil Code of the People’s Republic of China* (hereinafter referred to as the *Chinese Civil Code*), known as the “Encyclopedia of social life” has come into effect since January 1, 2021. It follows the civil law tradition, mainly based on German Civil Code [1, 2], for the lack of codified civil law in ancient China [3], leading to the fact that some legal terms are loaded with profound culture, especially after the reform and opening of China’s accession to the World Trade Organization. People without a legal background in Anglo-American countries have difficulties in understanding the connotations of these terms created by Chinese authorities or sourced from civil law tradition because these terms are gap or void in Anglo-American culture, which requires translators to accurately grasp the Skopos for choosing appropriate principles and take the equivalence of legal functions into account.

At the same time, another approach is applied as further clarification and supplement, namely Eco-translatology, proposed by Chinese scholar Hu Gengshen. Eco-translatology is an interdisciplinary study combining ecology and translation studies [4]. One of its basic ideas is “Translation as Adaptation and Selection”. Hu believes that the translation method of Eco-translatology is “multi-dimensional”, which is similar to the purpose of Skopos theory.

Under the principle of “multi-dimensional adaptive selection”, translation activities are selected and transformed at the level of language dimension, cultural dimension and communicative dimension. The language dimension, cultural dimension and communicative dimension are complementary and inseparable, and the higher the degree of integration, the more appropriate and effective the translation will be. The higher the degree of integration, the more relevant and effective the translation will be. Under the principle, translation activities are selected and transformed at the level of language, culture, and communication, which are complementary and inseparable. The higher the degree of integration is, the more appropriate and practical the translation will be.

Terminology is indispensable to science [5], expressing connotations of a theory [6] and manifesting essential features [7]. Especially, legal terms, which are tied to the legal system [8], provide multiple ways of structuring abstract legal knowledge across traditions and culture-bound singularity [9]. However, the complexity of law and the particularity of terminology [10] require term translation to adopt various principles and strategies. Term translation, a dual operation of language and legal transformation at the same time [11], should serve as the formation of equivalent terms representing the same concept in different languages [12]. However, due to the lack of equivalents of legal terms in different legal systems, the cultural traces in legal words cannot be fully transmitted [13], and the connotations deviate after transplantation [14], which are two significant difficulties encountered in comparative legal analysis [15, 16]. Therefore, this paper aims to establish equivalence in translation of legal terms [17] and probe into the factors characterized by the source-language culture, which are gaps or voids in the target language [18].

Scholars have made abundant research on equivalent translation in law. Vîlceanu Titela and Tanciu Maria Georgiana Stoenică study on linguistic and cultural mediation in legal translation [19]; Fernando Prieto Ramos gives an an empirical cross-genre study on why terminology is a difficulty in international legal document translation [20], and further researches on translating legal terminology and phraseology between inter-systemic incongruity and multilingual harmonization [7]; De Laforcade Agata analyzes linguistics difficulties in harmonisation of European Union Law under the example of Directives on Procedural Guaranties in Criminal Matters [21]; Zhang Falian builds the ethical theory of legal translation technology [22] and calls on the cultural confidence in the C-E translation of *Chinese Civil Code* to accurately contribute to transferring and publicizing the legal culture [23]; Zhao and Xue explore conceptual transplantation and equivalent interpretation in legal translation with terms of *Chinese Civil Code* [17]; Qu analyzes the principles of Chinese legislative text translation based on the English version of *Chinese Civil Code* [24] and puts forward the principle of equal authenticity different from the principle of equivalence [25]; Wu explores translation norms and strategies in legal terms in light of digital humanities [26]. Other scholars also pay attention to translation practice under EU legal terminology database [27, 28] and the properness of translation community [29].

Different from the above research, this paper focuses on three objectives: (1) how to select translation principles under the guidance of Skopos theory; (2) how to apply the principles in the translation of legal terms with Chinese characteristics; (3) what the relationships between principles, strategies, and term types are. This paper constructs a corpus of legal terms with Chinese characteristics which are classified into newly-coined and inherited terms. Based on various principles, the practical principles suitable for term translation will be concluded with several detailed examples for further illustration. Finally, the following parts will give the relationship between principles, strategies, and term types through the analysis of collective data.

## The Skopos theory

Translation of legal terms not only involves the expression of connotations contained in the source language but also is a creative activity to transform the information with ideological and cultural characteristics [30]. Since the correspondence between terms of two different languages is not identical to a great extent, certain factors (such as linguistic, cultural, or formal factors) are absent or impossible to retain in translation [31]. It is essential to clarify the target readers and then determine the translation style accordingly [32]. In the specification of transforming terms with Chinese characteristics and style, the target readers’ thinking paradigm and expression habits should be fully taken into consideration, and the application of the Skopos theory could reach the effects of functional equivalence.

The Skopos theory, developed by German translator Vermeer [33], is a new model of translation theory guided by human behavior, defining translation as a process of intercultural interaction with specific purposes and intentions. Under this theory, translation is purposeful behavior, and translators should determine the purpose according to the intended audience, considering their cultural background, expectations and communicative needs [34].

There are three rules in the Skopos theory. First, the Skopos rule is the primary one, which could be commonly explained that the initiator of the translation determines the Skopos [33]. The other two are the coherence rule and the fidelity rule. How to apply the fidelity rule depends on the Skopos of translation. When the Skopos requires the translation to be as close to the original text as possible, the translator will try his or her best to represent the characteristics, language style and contents of the source text. Meanwhile, the Eco-translatology shares a similar idea in translation, which is another type of guidance, from Chinese scholars’ perspective, in explaining translator behaviors.

In applying the Skopos theory, the Skopos is a critical point because it determines translation principles and rules. In the Skopos rule, the initiator, ideally based on his or her translation brief, will give the information, including the initiator of the translation, the communicative Skopos, the receiver of the text, the medium, and the intentions of receiving the text. Simultaneously, the translator, instead of passively accepting, can determine the purpose to a certain extent by negotiating with the initiator, especially when the latter lacks professional knowledge or does not know the purpose for some reason. According to the Skopos rule, we conclude the necessary information in Table 1.

**Table 1:** Information based on Skopos rules.

**The initiator of the translation**	Legislative Affairs Commission of the Standing Committee of the National People’s Congress
**The Communicative skopos**	The official English translation of the *Chinese Civil Code*, translated and reviewed by an expert group established by the Legislative Affairs Commission of the Standing Committee of the National People’s Congress, aims at explaining the Chinese civil methods in the new era to the world, and to share the new achievements of China’s rule of law construction with the world [35].
**The receiver of the text**	Foreigners
**The medium**	Newspaper, report, Internet, etc.
**The intentions of receiving the text**	Learn about the *Chinese Civil Code* and Chinese civil systems and ideas

The translation requirements above imply that the official translation of the *Chinese Civil Law* is a bridge for the Chinese government to communicate with other countries, and the ultimate purpose is to promote the publicity and exchange of China’s legal system, telling the story of China’s legislation [35]. The Chinese government has expectations that through the translation of the *Chinese Civil Law*, the world can view Chinese civil systems and policies in a correct, friendly, and cooperative way. China has found a development path suitable for its actual conditions, with unremitting efforts to create legal systems with Chinese characteristics. However, whether the purpose could be realized, many issues impede translation, such as linguistic differences, cultural diversity, different thinking modes, psychology and so forth. Therefore, the selected principles should be accepted by the target readers willingly.

## Research, methods and data

### Methods of translation principle selection

This study aims to appreciate and examine the strategies in the translation of legal terms with Chinese characteristics in *Chinese Civil Code*, centering on five principles, namely, accuracy, readability, traceability, conventionality, and unification based on the corpus. Three methods are used in this paper:

1. Combination of quantitative and qualitative analysis. This paper collects and transcribes a remarkable corpus of legal terms with Chinese characteristics in *Chinese Civil Code*, with detailed quantitative and comprehensive qualitative analysis. The selection criteria will be provided in the following section.
2. Combination of examples and illustration. Typical examples from the *Chinese Civil Code* are demonstrated with profound explanation in light of related principles and strategies of legal term translation.
3. Combination of theory and practice. Based on the Skopos theory, this paper summarizes five principles and strategies that can be applied in the *Chinese Civil Code* and makes a thorough inquiry about the primary principle and about how to deal with the conflict between the principles.

The essence of translation is to spread ideas on condition that translations of terms are correct [36]. In this sense, the term translation is the destination of all kinds of translation. Therefore, four principles as follows could help with term translation [37]. First, the principle of accuracy refers to fully and correctly conveying the connotations of terms. Second, the principle of readability aims at translating terms understandable in the style of the target language. Third, the principle of traceability enables readers to recognize the sources of terms [38]. According to Jiang [37], in the face of the term translation dilemma, the principle of conventionality makes translation follow the habits and traditions. However, Hou [5] disagrees with Jiang’s conclusion: accuracy should be the first while readability and traceability the second, and readability and traceability can even be discarded for higher accuracy. Other scholars point out that when discussing the term translation, the most important thing is to be concerned with the conventionality of the term [38, 39].

Scholars raise the main problems in the translation of legal terms. Firstly, the unification and standardization of terms have received insufficient attention [40]. Secondly, there is a shortage of specialization, especially reflected in translating legal terms into common expressions or colloquial speeches, failing to follow the principle of “translating terms with terms”. One main reason is that translators lack solid knowledge of Chinese law and Anglo-American Law. The third problem is that some translations are unsystematic because legal terms exist in relation, not in isolation. At the same time, compared with translation principles of Anglo-American legal terms, we should focus on the advancement of legal terms for the meaning is constantly developing. In addition, expression habits and language connotations should also be taken seriously.

While translating legal terms with Chinese characteristics, translators should pay more attention to word selection and fully consider the connotation and the impact it may bring, because any slight negligence would lead to severe consequences. This part will analyze how to choose translation principles in legal terms in the *Chinese Civil Law* based on the Skopos theory in the following three aspects.

1. According to the Skopos rule, the translation process, including the selection of translation methods and strategies, is determined by the purpose of translation. For cultural communication and academic exchange of the *Chinese Civil Law*, the translation should be faithful to the original text, and translators are not allowed to add, delete or change the order as they please because any nuance would lead to huge differences and violate the principle of accuracy. However, even though accuracy is of great significance, it cannot achieve the skopos alone because the publicity effect is based on not only accuracy but also understandable connotations.
2. According to the fidelity rule, translators should judge whether the form and function of the source text meet the target culture’s requirements according to the predetermined Skopos to achieve interlingual coherence. However, it does not mean that translators should sacrifice everything for accuracy and cannot delete the repetitive expressions or even choose free translation. Translators could adopt the method of interpreting the connotation for higher readability; they could also follow the convention or make target readers identify the source of the term by literal features, which is the main content of the principle of conventionality and traceability.
3. According to the coherence rule, translations should meet the standards of intralingual coherence and be meaningful in the target language. Therefore, due to the connotation and serious political function of legal terms with Chinese characteristics, we cannot completely rely on foreign media or translators, leading to the principle of unification which requires the authorities to create formal translations and keep consistent in official documents.

All in all, five principles are concluded in the translation of legal terms with Chinese characteristics: accuracy, readability, traceability, conventionality, and unification.

### Data collection

This paper focuses on the translation principles of legal terms with Chinese characteristics. Since there are many terms sourced in Anglo-American law, translations would be better to abide by the expressions in a source law. Therefore, this paper selects 42 legal terms with Chinese characteristics in the *Chinese Civil Code* as the research object, which can be divided into two categories: first, newly-coined legal terms from the pioneering system in China; second, the inherited legal terms learned from the Roman or German Law. The selection criteria and reasons are as follows:

1. Newly-coined legal terms are created by the Chinese legislative authorities and do not exist in legal systems of other countries. Therefore, these terms deserve great attention because the Chinese government attempts to share the pioneering legislative experience with the world, in which the translation quality determines the effect of publicity. Besides, there are no translation conventions to follow.
2. As for inherited legal terms, they are transformed from Roman or German legal terms. These terms are easier for translators because they learn from experience of many countries in civil law systems.

In Table 2, most selected terms are from three parts: Book One, Book Two, and Book Four of *Chinese Civil Code*, for the following reasons:

1. Book One of General Part includes legal person system related to Chinese political systems, which creates special legal persons with Chinese characteristics, such as state organ legal persons, rural collective economic organization legal persons, urban and rural cooperative economic organization legal persons, and so on. Therefore, legal terms in Book One are newly-coined.
2. Book Two of Real Rights contains the content of the unique registration system of immovable properties, the ownership structure that can be divided into state ownership, collective ownership, private ownership, and original types of usufructuary rights, which are all created by China. In conclusion, these terms are mostly related to the land system, explaining the separation of buildings and land, land functions determined by urban or rural areas, and land owned by the state and collective [41, 42]. Many legal terms in this book are newly-coined and only a few originate from Roman and German law.
3. Book Four, recently independent of Book One of General Part, presents protection to personality rights and interests. These legal terms are shared and accepted worldwide, all inherited legal terms.

**Table 2:** Term distribution.

Book (Chapter)	Percentage
**Book One: General Part**	26%
**Book Two: Real Rights**	38%
**Book Three: Contracts**	7%
**Book Four: Personality Rights**	26%
**Book Seven: Tort Liability**	3%

This paper aims to divide selected legal terms into newly-coined and inherited terms, with the former referring to legal terms created by original Chinese systems and the latter to legal terms inherited, mainly from Roman law, according to China’s actual condition. Distribution could be seen in Table 3:

**Table 3:** Type distribution.

Type	Percentage
**Inherited Terms**	43
**Newly-coined Terms**	57

In handling legal terms with Chinese characteristics, translators should follow various translation strategies. Upon the corpus, this paper seeks to identify the relationship among strategies, as well as between strategies and legal term categories.

## Application of term translation principle in the *Chinese Civil Code*

### Principle of accuracy

A translated term should accurately convey the meaning of the original text. Otherwise, it would mislead the target readers. Accuracy, as the primary translation principle of “ABC” (Accuracy, Brevity and Clarity), should be considered more. Accuracy is of prime significance to the translation like a cornerstone to a building, and terms convey complex connotations and work as the foundation of the legal system, requiring more attention. It makes no sense if the translation conveys wrong meanings, no matter how elegant the translation is. In translating the *Chinese Civil Code*, the translator firstly comprehends the intention of the terminology, and tries to find the corresponding or similar terms in Anglo-American law, taking flexible means to seek common ground while reserving differences. The translation of “农村集体经济组织” is a good example for illustration.

“农村集体经济组织” *refers to, based on the Agricultural Cooperation Movement and the people’s commune, the three types of collective economic organizations which emerged since the reform and opening up, including town, village, and group* [43]. *Since the connotation of this term is identical to its literal meaning, the translator divides it into four parts to find the corresponding words of every part and combines them according to English habits, contributing to the final translation “Rural collective economic organization”*.

There are other examples, such as “不动产统一登记制度” into “Unified registration system with respect to immovable property”, “事业单位” into “Public institutions”, “国家所有权” into “State ownership”, “人格权” into “Personality right”, “不当得利” into “Unjust enrichment”, and “相邻关系” into “Adjacent relationships”. However, when legal terms have no corresponding concepts or notions in English, guided by the accuracy principle and the adaption approach proposed by Eco-translatology, the most appropriate and easy-to-understand words should be selected.

Theoretically, translators should take accuracy as the first and foremost principle to retain the original meaning. However, any process of pinpointing the corresponding expression in the target language is realized at the cost of losing a degree of accuracy, which is acceptable and reasonable. When translating other categories of terms, transliteration, interpretation, and colloquial expression are the final choice when there is no alternative. Another scholar points out that if there are no exact equivalents in Chinese, the translator can flexibly utilize functional equivalents, lexical expansion, paraphrasing, neutral terms, and borrowing on the premise of faithfulness [1]. However, the translation of legal terms, as a by-product of legal cultural exchange and transplantation [39], aims to be well comprehended by target readers. Transliteration is rarely used, and interpretation for functional equivalents would be advantageous for target readers to understand Chinese legal culture.

Similarly, Eco-translatology requires translators to make adaptive transformations of linguistic forms. This adaptive selection of linguistic dimensions for conversion is carried out in different aspects and at different levels. Chinese and English belong to different language families with remarkable differences. For example, English focuses on morphological fit, and the logic and structure between statements are often expressed through prepositions and clauses, etc. However, Chinese is an intentional spiral language, and logic is often expressed through context, chronological order, or sequence of event development.

### Principle of readability

Together with accuracy, terms should be translated comprehensibly and understandably, which requires the precise positioning of the target readers, in order to spread ideas and cultures. However, when accuracy and readability cannot coexist, the former should be put in the first place, or readable translation without accuracy would inevitably overshadow the brilliance of the writer and become a recreation of the translator [37].

Therefore, in translating legal terms, readability of translation should be promoted on the foundation of high accuracy. Moreover, terms can be divided into two types. The first is terms with precise literal meanings, which can be translated word by word. For example, the word “国有财产” is converted into “The state-owned property” in English. The other is terms with complex connotation, difficult to be understood literally, which could be translated freely under the Skopos theory.

*With strong nationality, this concept of* “宅基地” *does not exist in traditional civil law countries, and is the first independent institutional arrangement in the Chinese Property Law of 2007* [3]. *On the basis of collective membership*, “宅基地” *refers to farmers’ obtaining rural land free of charge to build houses, which is a welfare security system* [6].

*In the translation of* “宅基地使用权,” *alien from* “农村集体经济组织” *that can be translated word by word, free translation is the best choice but should be applied under the Skopos theory. It is translated into “Right to use a house site”, in accordance with the Skopos rule* [33] *as follows*:

1. *A translation (or TT) is determined by its Skopos of spreading cultures and ideas. The official translation gives a concise definition, which can be unquestionably comprehended by English natives (also the target readers)*.
2. *The top-ranking rule refers to the fact that the translation behavior is determined by its Skopos, which can also be interpreted as “the end justifies the means”. In this regard, the translator chose free translation instead of word-by-word translation or transliteration*.
3. *The fidelity rule requiring that translation should be as faithful as possible to the original texts, is fully abided by the official translation by emphasizing two points. First, “the right to use”, rather than “the right to own”, reveals the special system of separation of houses and lands and lands owned by state and collectives; second, the application of “a house site”, other than “a building site”, indicates that the purpose is limited to building a house for a living, not for commercial use*.

Sometimes, although literal translation meets the requirements of accuracy, it will make target readers have difficulty in distinguishing one term from another, for example:

“身体权” *and* “健康权” *are indistinguishable, and if they are translated into “Right to body” and “Right to health,” the target readers would be confused about the differences between “body” and “health,” considering the two terms are similar in meanings. However, in Chinese law*, “身体权” *is a right to protect the integrity of a person’s body, while* “健康权” *aims to protect the normal functioning of body tissues and organs. Therefore*, “身体权” *is officially translated into “Right to corporeal integrity” for better differentiation. Generally, these translations have enhanced the readability of legal terms, which is in line with the principles in the Skopos theory and Eco-translatology. Therefore, the translator should pay more attention to the transmission and interpretation of bilingual cultural connotations for better communicative demands*.

### Principle of traceability

Traceability enables the readers to easily identify the source of the translated terms and enjoy the characteristics of etymological language. However, if traceability is applied separately with accuracy and readability in term translation, readers might be confused by new terms, and cultural exchange will be impeded as a result.

*For example*, “准合同” *properly deals with all kinds of legal obligations, such as unjust enrichment and management without cause. The term* “准合同” *possesses the corresponding concept in Anglo-American law and French law* [32], *tracing back to the “Quasi-contracts” in Roman law. Therefore, the official translation follows the concept in Roman law to make the target reader recognize the origin at a glance*.

“居住权” *originated from the concept “habitatio” of Roman law, which is the right of non-owners to live in houses owned by others* [44]. *The official translation “The right of habitation” shows its etymological characteristics*.

There are pioneering undertakings in system designs of the *Chinese Civil Code*, which is a great initiative combined with China’s national conditions and Roman law. Therefore, accuracy can be ensured by citing the original expression in translations.

However, through comparing the English translation of the legal terms in civil law countries, although the inheritance relationship exists, there are many different expressions. Most English-speaking countries belong to the common law system. Therefore, when translating the terms in the civil law system into English, translators should first sort out the evolution of a term in civil law system, compare it with the similar concept in the common law system, and eventually find out the translation methods that can achieve equal interpretation between different legal systems [17].

“不动产” *is translated into “Land” in German Civil Code, “Real estate” in Japanese Civil Code, while “Immovable property” in Chinese. However, in Anglo-American law, “Real property” refers to itself or the rights on the real estate, obviously different from* “不动产” *in the Chinese Civil Law. However, the word “Immovable” is generally used in the legislative texts and legal works of Anglo-American countries, manifesting that “Immovable property” is suitable in both Chinese and the target language*.

### Principle of conventionality

This principle means that in the face of different translations of a term, interference and unification are unnecessary, especially the administrative measures, because the language has its own rules. Democratic discussion and selection truly ensure the quality of term-translations.

*In the translation of* “无因管理” *into “Negotiorum gestio”, obviously abiding by the tradition of Roman Law and German Law, is the best translation chosen by history and is familiar to many scholars even though it lacks readability to a certain extent*.

The word “居住权” is translated into “The right of habitation”, which also embodies the principle of conventionality. Also, “法人” into “legal person” is in line with the convention of Anglo-American law.

### Principle of unification

The Chinese-English translation of legal terms with Chinese characteristics lacks unity and changes at will, negatively impacting the quality of international communication in Chinese law. The principle of unification has the following requirements: (1) the unified translation shall be published by the highest authority or media; (2) when there are multiple versions published by the highest authorities, the latest one shall be taken as final [40].

However, the principle of unification is not rigid or universal, considering that some legal terms are cross-category with two or more functions (such as both nouns and verbs). Moreover, it is natural that there are various translation methods under the condition that the core part remains unchanged.

“集体所有权” *is officially translated into “Collective ownership”, in which the word “collective” is a cross category word, both as a noun and an adjective*.

### Literal or free translation

In the *Chinese Civil Code*, 21 newly-coined terms are translated word by word, while some are free translated, including “农村承包经营户” into “Rural usufructuary households”; “预告登记” into “Registration of a priority notice”, and “宅基地使用权” into “Right to use a house site”, the inner connotation of which are difficult to be recognized literally. If these terms are translated word by word, the purpose of cultural communication and academic exchange would be impeded, fully violating both the Skopos and the fidelity rule.

*In the translation of* “预告登记”, “预告” *is abstract, which means the obligee of a house can register before he or she owns the house. Its function is to protect the obligee, preventing the developers from selling the house to others, causing infringement to the obligatory right. The authority chooses “a priority notice”, meaning that there is a notice that someone has bought the house and owns the obligatory right when the second buyer registers the house, perfectly manifesting the connotation of the legal term by free translation*.

However, for the rest of the legal terms, although they could be translated literally, the translation should also be guided under the Skopos theory. Therefore, the translation should not only rely on these terms to convey the literal meaning to meet the skopos rule and fidelity rule, but abide by the coherence rule and the principle of unification, because it is not a series of separate processes, but a closely integrated project. The principle of unification requires not only the standardization of translated terms across sectoral laws, but also the unification of terms in a code. In this way, although most newly-coined terms are translated word by word, they are still in line with unification in core parts. There are 18 inherited terms translated word by word for the same reason in our corpus.

*For example*, “权” *is translated into “right of” or “right to”, such as in “Right to contractual management of land”, “Right to management of land”, and “Right to use land for construction purposes”*. “农村”, *as an adjective, is translated into “rural”, such as in “Rural usufructuary households”, “Rural collective economic organization legal persons”, and “Urban and rural cooperative economic organization legal persons”, embodying the principle of unification*.

## Conclusion

This paper makes a comprehensive analysis of translation principles and strategies in translating legal terms with Chinese characteristics in the *Chinese Civil Code*. This research is innovative in three aspects. First, the research objects are collected from the official translation of the *Chinese Civil Code*, which is recently published and lacks systematic research. Second, this paper selects legal terms with Chinese characteristics instead of all terms in the *Chinese Civil Code* because many terms, especially in Book Three of Contracts, originate from Anglo-American law and have corresponding English translations, where there is no space for exploration. However, it is essential to make a thorough investigation on the translation as well as acceptance by target readers when there is no appropriate English equivalents and related legal systems. Third, scholars have done research on the translation of legal terms without definitive conclusion having been reached due to the different content, form, audience, and function of the laws of various departments. At the same time, this paper applies the Skopos theory in analyzing translation principles and explores how these principles work and how to deal with the conflicts among principles.

Through the investigation and analysis of the corpus above, this paper focuses on three research questions:

1. How to select translation principles under the Skopos theory.
  In light of the Skopos theory, the official translation of the *Chinese Civil Code* is a bridge for cultural communication and academic exchange. Therefore, translators should follow the principle of accuracy, and translations should convey the information of the original text to a large extent. According to the fidelity rule, translations should lay emphasis on readability through taking the habits of the target language into account. The principle of traceability enables readers to easily identify the source of the term with characteristics of the etymological language maintained. The principle of conventionality is beneficial to widely-accepted translation. Through the coherence rule, the translation could meet the standards of intralingual coherence and the principle of unification, not only among the terms, but the core parts of terms. At the same time, this paper applies the Skopos theory and Chinese translation theory of Eco-translatology in analyzing translation principles and explores how these principles work and how to deal with the conflicts among these principles.
  In general, translators should give full consideration to their subjectivity and make adaptive choices in both translation objects and operations. When the source language and the target language cannot be optimally converted in the same dimension, inter-dimensional conversion is indispensable. Under the guidance of both the Skopos theory and Eco-translatology, translators should use appropriate strategies to improve the quality and communication effectiveness of translations to convey Chinese wisdom to the international community.
2. How to apply the principles in translating legal terms with Chinese characteristics.
  This paper makes a detailed analysis of applying these selected translation principles and dealing with the conflicts among principles. In the principle of accuracy, the translator should firstly comprehend the intention of the terminology, and try to find the corresponding or similar terms in Anglo-American law. The reasonable loss of accuracy is acceptable for translation is a by-product of legal and cultural exchange. Finally, flexible means should be made to seek common ground while reserving differences, such as interpretation and colloquial expression. As for the principle of readability, it requires the translation to spread ideas and cultures in an appropriate way of the target groups. However, when accuracy and readability conflict, the readability of translation should be based on the foundation of accuracy. The term with clear literal meanings can be translated word by word, while those of difficulty in understanding literally should be solved through free translation under the Skopos theory. For the principle of traceability, it cannot be applied in term translation separately with accuracy and readability, and for inherited terms, translators should first sort out the evolution of the term in the civil law system, compare it with the similar concept in the common law system, and eventually find out the translation methods that can achieve equal interpretation between different legal systems. For conventionality, the democratic discussion and selection truly ensure the quality of term translations, and the translators should respect the convention. For the principle of unification, the unified translation shall be published by the highest authority or media. However, the principle of unification is not rigid or universal, and two or more methods of translation can exist if the core part remains unchanged, considering that some legal terms are cross-category words.
3. The relationship between principles, strategies, and term types.
  In the five principles, accuracy is primary, and the other four principles should abided by the principle of accuracy.

This paper also analyzes the translation strategies and their relationship with legal term categories. Newly-coined terms, the connotations of which cannot be easily understood literally, should be free translated under the principle of accuracy and the Skopos and fidelity rule. In contrast, others should be translated word by word under the principle of unification and the coherence rule.

## Supporting information

S1 Data (XLSX)

S1 File (PDF)
