# Supplementary material for: Translation strategy of legal terms with Chinese characteristics in Civil Code of the People’s Republic of China based on Skopos theory
Source: PLoS One. 2022 Sep 22;17(9):e0273944. doi: 10.1371/journal.pone.0273944 (PMC9498954; doi:10.1371/journal.pone.0273944)
Supplement: S1 File — (PDF) [file pone.0273944.s002.pdf]

Legal terms used in this article are attached to Table 4

Table 4. The distribution and classification of legal terms with Chinese characteristics in the original book

Terms	Public Translation	Book	Catergory
个体工商户	Individual industrial and commercial households	One	New
农村承包经营户	Rural usufructuary households	One	New
事业单位	Public Institutions	One	New
社会团体	Social groups	One	New
社会服务机构	Social service organizations	One	New
捐助法人	Donation-based legal person	One	New
特别法人	Special legal persons	One	New
机关法人	State organ legal persons	One	New
农村集体经济组织法人	Rural collective economic organization legal persons	One	New
城镇农村的合作经济组织法人	Urban and rural cooperative economic organization legal persons	One	New
基层群众性自治组织法人	Basic self-governing mass organization legal persons	One	New
不动产统一登记制度	Unified registration system with respect to immovable property	Two	New
不动产登记簿	The register of immovable property	Two	New
不动产权属证书	The real right certificate for immovable property	Two	New
预告登记	Registration of a priority notice	Two	New
国有财产	The state-owned property	Two	New
集体财产	The property owned by a collective	Two	New
建筑物区分所有权	Ownership of a building’s units	Two	Inheritied
相邻关系	Adjacent relationships	Two	Inheritied
土地承包经营权	Right to contractual management of land	Two	New
土地经营权	Right to management of land	Two	New
建设用地使用权	Right to use land for construction purposes	Two	New
宅基地使用权	Right to use a house site	Two	New
居住权	Right of habitation	Two	Inheritied
国家所有权	State ownership	Two	New
集体所有权	Collective ownership	Two	New
私人所有权	Private ownership	Two	New
准合同	Quasi-contracts	Three	Inheritied
不当得利	Unjust enrichment	Three	Inheritied
无因管理	Negotiorum gestio	Three	Inheritied
人格权	Personality Rights	Four	Inheritied
生命权	Rights to life	Four	Inheritied
身体权	Rights to corporeal Integrity	Four	Inheritied
健康权	Rights to health	Four	Inheritied
姓名权	Rights to name	Four	Inheritied
名称权	Rights to entity Name	Four	Inheritied
肖像权	Rights to likeness	Four	Inheritied
名誉权	Rights to reputation	Four	Inheritied
荣誉权	Rights to honor	Four	Inheritied
隐私权	Rights to privacy	Four	Inheritied
个人信息保护	Protection of personal information	Four	Inheritied
侵权责任	Tort liability	Seven	Inheritied
